# Metallothioneins regulate the adipogenic differentiation of 3T3-L1 cells via the insulin signaling pathway

**DOI:** 10.1371/journal.pone.0176070

**Published:** 2017-04-20

**Authors:** Yoshito Kadota, Yuriko Toriuchi, Yuka Aki, Yuto Mizuno, Takashige Kawakami, Tomoko Nakaya, Masao Sato, Shinya Suzuki

**Affiliations:** Faculty of Pharmaceutical Sciences, Tokushima Bunri University, Tokushima, Japan; INIA, SPAIN

## Abstract

Knockout of metallothionein (MT) genes contributes to a heavier body weight in early life and the potential to become obese through the intake of a high fat diet (HFD) in mice. It has thus been suggested that MT genes regulate the formation of adipose tissue, which would become the base for later HFD-induced obesity. We evaluated the fat pads of mice during the lactation stage. The fat mass and adipocyte size of MT1 and MT2 knockout mice were greater than those of wild type mice. Next, we assayed the ability of small interfering RNA (siRNA) to silence MT genes in the 3T3-L1 cell line. The expressions of MT1 and MT2 genes were transiently upregulated during adipocyte differentiation, and the siRNA pretreatment led to the suppression of the expression of both MT mRNAs and proteins. The MT siRNA promoted lipid accumulation in adipocytes and caused proliferation of post-confluent preadipocytes; these effects were suppressed by an inhibitor of phosphatidylinositol 3-kinase (LY294002). In addition, MT siRNA promoted insulin-stimulated phosphorylation of Akt, a downstream kinase of the insulin signaling pathway. Enhanced lipid accumulation in 3T3-L1 cells resulting from MT-gene silencing was inhibited by pretreatment with an antioxidant, *N*-acetylcysteine, used as a substitute for antioxidant protein MTs. These results suggest that interference in MT expression enhanced the activation of the insulin signaling pathway, resulting in higher lipid accumulation in 3T3-L1 adipocytes.

## Introduction

Metallothioneins (MTs) are low-molecular-weight and cysteine-rich proteins that play a regulatory role in the metabolism of essential metals such as zinc and copper. These proteins also play preventive roles in protection against damage associated with heavy metal toxicity caused by cadmium and mercury, oxidative stress, and endoplasmic reticulum (ER) stress.[1–3] MTs are induced by various stimuli including metals, reactive oxygen species (ROS), inflammatory cytokines, and hormones.[4] There are at least four subfamily members in mammals: MT1, MT2, MT3, and MT4. The cluster of MT genes is located on chromosome 16 in humans and on chromosome 8 in mice. Human MT1 and MT2 genes have functional subisoforms (MT1A, MT1B, MT1E, MT1F, MT1G, MT1H, MT1M, MT1X, and MT2A).[5] Whereas MT1 and MT2 are expressed ubiquitously and these proteins are thought to be functionally equivalent, MT3 is mainly expressed in the brain, and MT4 is specifically expressed in squamous epithelium of the skin and tongue.[6–8]

Many common polymorphisms have been found in the human MT gene region.[9] Polymorphisms of the MT1A, MT1B, and MT2A genes have been significantly associated with metabolic abnormalities including type 2 diabetes mellitus and hyperlipidemia.[10,11] Thus, MTs may play preventive roles in the protection against metabolic disorders, and polymorphisms can affect the protein functions and/or the expression level of MTs. However, it is unclear whether MTs protect against obesity-associated complications and metabolic disorders.

Obesity is associated with an increased risk of developing insulin resistance and type 2 diabetes. The development of obesity consists of 3 processes: differentiation of preadipocytes into mature adipocytes, an increase in the number of fat cells (hyperplasia), and an increase in the size of adipocytes caused by excess accumulation of lipids in fat cells, which results in cell enlargement (hypertrophy).[12] The number of adipocytes increases in early life (childhood and adolescence) during white adipose tissue (WAT) development, whereas the amount is usually constant in adults.[13] Obesity beginning in childhood is characterized by hyperplasia and hypertrophy, and adult-onset obesity is characterized by hypertrophy. MT1, MT2, and MT3 express in adipose tissue [14], and the expression of MT1 and MT2 in rodents is affected by nutritional status [15]. We previously reported that female MT1 and MT2 knockout (MTKO) mice developed high-fat-diet (HFD)-induced obesity, but that a standard diet did not induce obesity in the mice.[14,16] In addition to MTKO mice, Lindeque *et al*. showed that MT3 KO mice significantly increased their body weight through the intake of a HFD compared with wild type mice.[17] Male hybrid MTKO mice (129/Ola and C57BL/6J background) and MT3 KO (C67/BL6 and 129/Sv background) exhibited obesity.[18,19] Interestingly, these KO mice already had a higher body weight than the wild type (WT) mice at age 3–4 months old, i.e., prior to HFD feeding.[17] Therefore, MTs may be involved in the regulation of the number of adipocytes and WAT formation at earlier stages, which would contribute to later obesity induced by HFD feeding.

In this study, we investigated the effects of the decreased expression of MT caused by siRNA on adipocyte differentiation, and we discuss the possibility that MTs regulate adipose development.

## Materials and methods

### Materials

3-isobutyl-1-methylxanthine (IBMX), H89 dihydrochloride hydrate and Oil Red O were purchased from SigmaAldrich (St. Louis, MO, USA). RNAiso Plus and NucleoSpin RNA were obtained from Takara Bio (Otsu, Japan).

### Animals

Five-day-old WT and MTKO mice (129S7/SvEvBrd-*Mt1*^*tm1Bri*^ *Mt2*^*tm1Bri*^/J), which were developed by Masters *et al*.[20], and wild type mice (WT, 129S1/SvImJ) were purchased from Jackson Laboratory (Bar Harbor, ME, USA). These mice were maintained under specific pathogen-free conditions and mated only with the 129 mouse strain to maintain the genetic background. These mice were housed in a temperature-controlled room at 22°C with 55±5% humidity under 12-h light/dark cycles. The female mice were fed the chow diet (NMF; Oriental Yeast, Tokyo, Japan) and were watered *ad libitum* during pregnancy and lactation. All experimental procedures were approved by the Animal Care and Use Committee of Tokushima Bunri University and conformed to the guidelines of the Japanese Ministry of Education, Culture, Sports, Science, and Technology.

### Histology and analysis of WAT weight

Five-day-old WT and MTKO mice were sacrificed by blood removal and cervical dislocation under isoflurane anesthesia, and their dorsal-ventral subcutaneous fat pads were excised and weighed. All efforts were made to minimize suffering. The percent WAT weight was calculated by dividing the fat mass by the body weight. WAT samples collected from the mice were fixed in Mildform 15NM (Wako Pure Chemicals, Tokyo, Japan) and embedded in paraffin. WAT sections (3 μm thick) were prepared on silane-coated slides and stained with hematoxylin and eosin (HE). Digital images of the WAT sections were obtained using a Leica DMLA microscope with a CCD camera (Leica, Wetzlar, Germany).

### Cell culture and induction of adipocyte differentiation

All cells were cultured in a humidified atmosphere at 37°C with 5% CO_2_ and 95% room air. Mouse 3T3-L1 preadipocytes were purchased from DS Pharma Biomedical Inc. (Osaka, Japan). The protocol used to induce the differentiation of 3T3-L1 preadipocytes into adipocytes was adapted from the method presented by Reed and Lane.[21] 3T3-L1 cells were seeded at a density of 3 × 10^5^ cells/well on a 6-well plate in 2 ml of Dulbecco's Modified Eagle Medium (DMEM) containing 10% calf serum (CS) and precultured for two days. After the preculture, adipogenic differentiation of preadipocytes into adipocytes was induced on day 0 by replacing the original medium with DMEM containing 10% fetal bovine serum (FBS) supplemented with 1 μg/ml insulin, 1 μM dexamethasone (DEX), and 0.5 mM IBMX, which was referred to as differentiation- inducing medium (DIM). Two days after stimulation of differentiation (day 2), the culture medium was changed to DMEM containing 10% FBS supplemented with 1 μg/ml insulin, and cells were cultured for two additional days. On day 4, the medium was replaced with DMEM containing 10% FBS. The medium was changed every two days during all culture stages. Each inhibitor (50 μM PD98059, a mitogen-activated protein kinase kinase (MAPKK) inhibitor; 50 μM LY294002, a phosphoinositide 3-kinase (PI3K) inhibitor; 10 μM H89, a protein kinase A (PKA) inhibitor) or dimethyl sulfoxide (DMSO) as a vehicle control was added 30 min prior to preadipocytes that received DIM treatment for 24 h. Preparation and culture of adipose-derived stromal cells (ADCs) was performed as previously described. [16] Briefly, murine fad pads were obtained from 5-day-old WT and MTKO mice. The adipose tissues were digested by collagenase. Isolated adipocytes were de-differentiated via ceiling culture into ADCs in T-25 Flasks (Nunc, Rochester, NY, USA) completely filled with DMEM containing 10% CS. ADCs from more than 10 passages were used.

### Pretreatment of small interfering RNA

The siRNA pretreatment, used to silence MT expression, was performed 24 h after cell seeding. MTs siRNA (metallothionein siRNA (m)) or control siRNA (control siRNA-A) (Santa Cruz Biotechnology, Dallas, TX, USA) (final conc. 50 nM) in Opti-MEM I (Life Technologies, Carlsbad, CA, USA) was transfected into the cells using Lipofectamine RNAiMAX Reagent (Life Technologies). After siRNA pretreatment for 24 h, the medium was removed, and the cells were used for the following assays.

### Oil red O staining

Lipid accumulation was evaluated by Oil Red O retention. The cells were fixed with 4% paraformaldehyde and stained with 3 mg/ml Oil Red O in 60% isopropyl alcohol. To quantify the retention of Oil Red O, the absorbance was measured at 520 nm using a microplate spectrophotometer Infinite 200 PRO (Tecan, Männedorf, Switzerland).

### RNA isolation and mRNA expression analysis

Cell lysates were prepared using the RNAiso Plus reagent (TAKARA Bio, Ohtsu, Japan). After the cells were lysed, chloroform (1/5 of the volume of the RNAiso Plus reagent) was added to the samples. The samples were centrifuged (11,000 × g) at 4°C for 15 min, and the supernatants were collected, to which 1 volume of RNAase-free water and 0.7 volume of ethanol were added. The lysates were then added to the Nucleospin RNA Kit (TAKARA Bio) columns, and total RNAs were purified according to the manufacturer’s instructions. For reverse transcription (RT), 2 μg of RNA from each sample was reverse-transcribed with a High-Capacity cDNA RT Kit (Life Technologies). For the real-time PCR, target genes were amplified with SYBR Premix Ex Taq II using a LightCycler 1.5 (Roche Applied Sciences, Basel, Switzerland). The selected primer sets used in the RT-PCR assay can be found as S1 Table. The PCR program was 95°C for 30 s, followed by 40 cycles of 95°C for 5 s, 55°C for 30 s, and 72°C for 30 s. To verify specificity, melting curve analysis and agarose gel electrophoresis were carried out on the real-time RT-PCR products. The relative amount of 36B4 mRNA, a gene whose expression in 3T3-L1 cells is unaffected by adipogenesis [22], as an internal control housekeeping gene was determined to compensate for variations in RT-PCR efficiency.

### Immunocytochemistry

3T3-L1 cells were seeded at 3 × 10^4^/well on an 8-well chamber slide. After various treatments, they were fixed with 4% paraformaldehyde (PFA) for two hours at room temperature. After washing with phosphate-buffered saline (PBS), blocking buffer (5% goat serum and 1 mg/mL bovine serum albumin in PBS) was added, and the cell specimens were incubated at 4°C overnight. Samples were then treated overnight with a primary antibody (anti-metallothionein mouse monoclonal antibody [UC1MT] (Abcam, Cambridge, UK); 1:100 dilutions in the blocking buffer) at room temperature. After washing in PBS with 0.05% Tween 20, a secondary antibody (Goat anti-Mouse IgG (H+L) Secondary Antibody, Alexa Fluor 488 (Catalogue #A-11001) (Life Technologies), 1:1000) was added. Hoechest 33258 was used for nuclear staining. Immunofluorescent images were captured using a BIO-REVO BZ-9000 fluorescence microscope (Keyence, Osaka, Japan).

### Bromo-2'-deoxy-uridine labeling

Proliferating cells were detected with Bromo-2'-deoxy-uridine Labeling and Detection Kit I (Roche Applied Sciences) according to the manufacturer’s protocol. 3T3-L1 cells were seeded overnight at 3 × 10^4^/well on an 8-well chamber slide. After siRNA or control siRNA pretreatment for 24 h, the confluent cells were treated or not treated (0 hour) with DIM for 24 h, after which 10 μM bromo-2'-deoxy-uridine (BrdU) in DMEM and 10% FBS was added for 2 h. Then, the cells were fixed with ethanol at -30°C in a freezer overnight. BrdU incorporation was detected using an anti-BrdU antibody and fluorescein-conjugated anti-mouse Ig antibody. All nuclei were counterstained with Hoechest 33258. Immunofluorescent images were captured using a BIO-REVO BZ-9000 or BZ-X700 fluorescence microscope (Keyence). The number of BrdU- or Hoechest-positive cells in 25 fields per well were counted using BZ-II Analyzer software (Keyence). The proportion of BrdU-positive cells in 3 independent wells was calculated and averaged.

### Sample preparation and western blotting

The cells were immediately harvested and sonicated in cold mammalian protein extraction reagent (M-PER; Pierce Biotechnology, Rockford, IL, USA) in which a cOmplete mini protease inhibitor cocktail tablet and a PhosSTOP phosphatase inhibitor cocktail tablet (Roche Applied Sciences) were dissolved. Adipose tissues were homogenized in cold-Tissue-Protein Extraction Reagent (T-PER; Pierce Biotechnology) in which cOmplete mini and PhosSTOP tablets were dissolved. Then the extracts were centrifuged at 15,000 × g for 15 min at 4°C. Aliquots of the resultant supernatants containing 20 μg of total proteins were treated with 25 mM mercaptoethanol and 167 mg/mL sodium dodecyl sulfate (SDS) and boiled at 100°C for 2 min. Then, each sample was subjected to SDS–polyacrylamide gel electrophoresis (SDS-PAGE) on a 10 or 12% acrylamide gel, followed by electroblotting onto polyvinylidene difluoride (PVDF) membranes using the Mini Trans-Blot Electrophoretic Transfer Cell (Bio-Rad Laboratories, Hercules, CA, USA). The membranes were treated with a 1:1000 dilution of rabbit antibodies against peroxisome proliferator-activated receptor γ (PPARγ) (polyclonal, #H-100; Santa Cruz Biotechnology, Santa Cruz, CA, USA), β-actin (polyclonal, #ab8227; Abcam, Cambridge, UK), RPLP0, also known as 36B4 (polyclonal, #11290-2-AP; Proteintech, Chicago, IL, USA), CCAAT-enhancer-binding protein β (C/EBPβ) (LAP) (polyclonal, #3087), C/EBPδ (polyclonal, #2318), FABP4 (polyclonal, #2120), fatty acid synthase (FAS) (monoclonal, C20G5), Akt (monoclonal, 11E7), phosphorylated Akt on Thr308 (monoclonal, C31E5E), or phosphorylated Akt on Ser473 (monoclonal, 193H12) (Cell Signaling Technology, Danvers, MA, USA) for primary antibodies, followed by treated with a 1:2000 dilution of anti-rabbit IgG, horseradish peroxidase (HRP)-linked antibody (#7074; Cell Signaling Technology) for secondary antibodies in Tris-based saline (TBS)-0.05% Tween 20. Proteins on the membrane were visualized using an Image Analyzer LAS-4000 (Fujifilm, Tokyo, Japan) and a chemiluminescent substrate (Millipore, Billerica, MA, USA). The intensities of immunoreactive bands were quantified using image analysis software (Multi Gauge V3.0, Fujifilm). Prestained SDS-PAGE standard (Bio-Rad) and a biotinylated protein ladder detection pack (Cell Signaling Technology) were used as molecular mass standard proteins to calculate the molecular weight of the proteins.

### Measurement of intracellular ROS level

3T3-L1 cells were seeded at 1 × 10^4^/well in 96-well black MicroWell plates with a Nunclon Delta (Nunc). Intracellular ROS levels were measured using 2´, 7´-dichlorodihydrofluorescein diacetate (DCFH-DA; Life Technologies). The cells were incubated in the presence of 10 μM DCFH-DA in FluoroBrite DMEM (Life Technologies) in a CO_2_ incubator for 30 min. After the medium was removed, the cells were washed twice with PBS and then were fixed with 4% PFA at RT in the dark. All nuclei were counterstained with 10 μg/mL Hoechst 33258 (excitation at 350 nm; emission at 461 nm) in PBS for 30 min. The cells were washed twice with PBS, and PBS was then added to each well. The values of fluorescent 2´, 7´-dichlorofluorescein (DCF), converted from DCFH-DA, were measured by excitation at 488 nm and emission at 530 nm, using a microplate spectrophotometer Infinite 200 PRO. The intracellular ROS levels were calculated from the ratio of DCF fluorescence to Hoechst fluorescence.

### Statistical analysis

The results are presented as the mean ± SD. Ekuseru-Toukei 2010 for Windows (Social Survey Research Information, Tokyo, Japan) was used for statistical analysis. Datasets were compared for significant differences using a paired Student’s *t* test or one-way analysis of variance followed by the Tukey-Kramer test or Dunnett’s test.

## Results

### Adipose tissue development in the lactation stage of WT and MTKO mice

**Fig 1:**
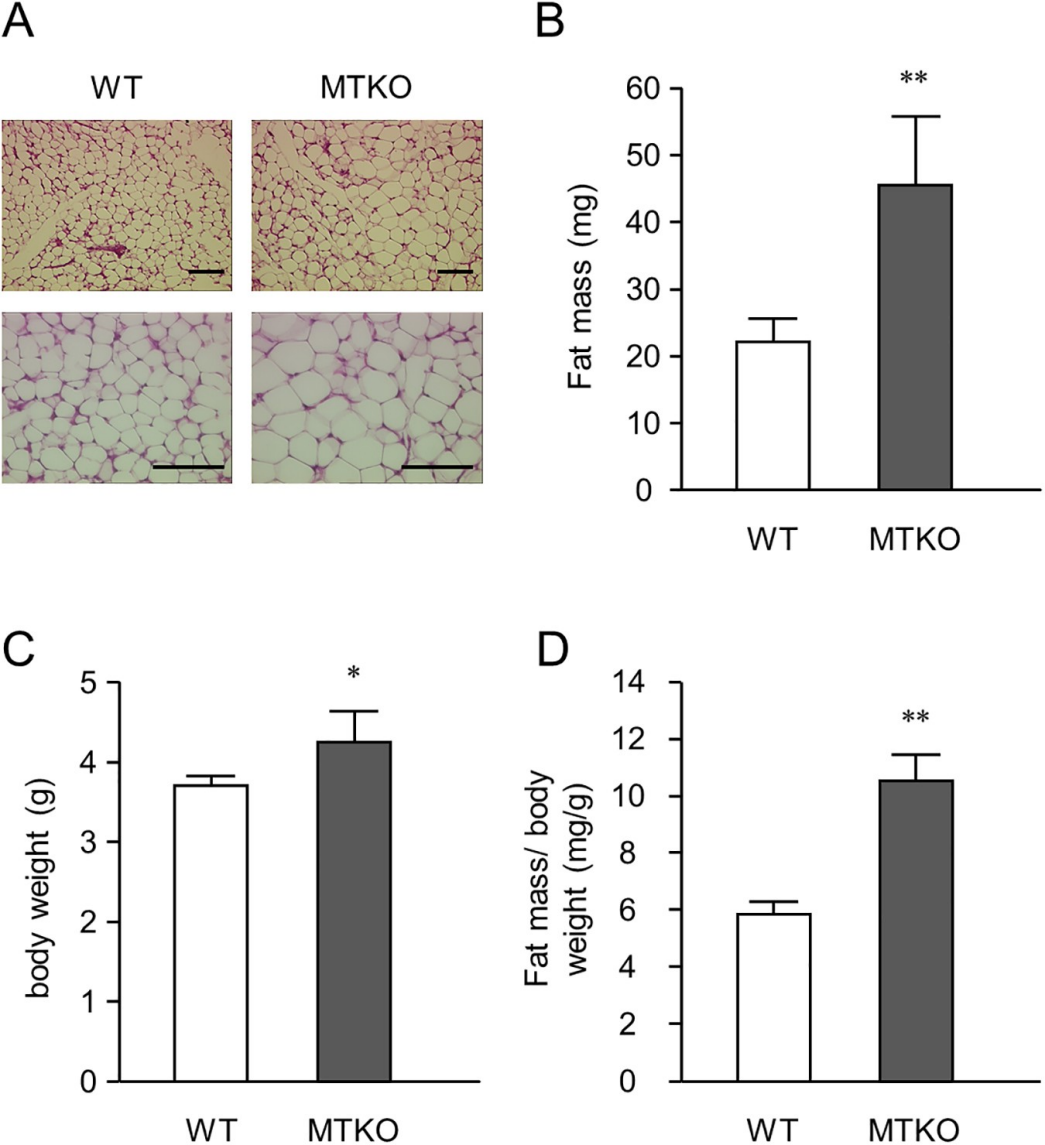
White adipose tissues (WATs) of five-day-old wild type (WT) and MTKO mice. Dorsal-ventral subcutaneous fat pads were excised from five-day-old mice. (A) Representative histology images of WAT in five-day-old WT and MTKO mice. WATs were larger in MTKO mice than in WT mice. Bar = 100 μm. (B) WAT weight and (C) body weight of five-day-old WT and MTKO mice. (D) Percent WAT weight was calculated by dividing fat mass by body weight. Values are presented as the mean ± SD (*n* = 5). ** represents a *P* value of < 0.01.

We first investigated whether MT1 and MT2 deficiency enhanced WAT formation and development. Immediately after birth, WATs in the 129/Sv strain were too small to identify by gross observation; however fat pads were developed and observable during the lactation stage. As shown in Fig 1A, adipocytes derived from the dorsal-ventral subcutaneous fad pads of suckling infant MTKO mice were larger than those of WT mice. The fat mass of the infant MTKO mice was markedly greater than that of WT mice (Fig 1B), and their body weight was slightly higher (Fig 1C). The ratio of WAT weight to body weight was also significantly greater in MTKO mice than WT mice (Fig 1D). These results suggest that MTs play a role in appropriately limiting WAT development, at least during the lactation stage.

### Effect of MTs siRNA pretreatment on lipid accumulation in 3T3-L1 adipocytes

**Fig 2:**
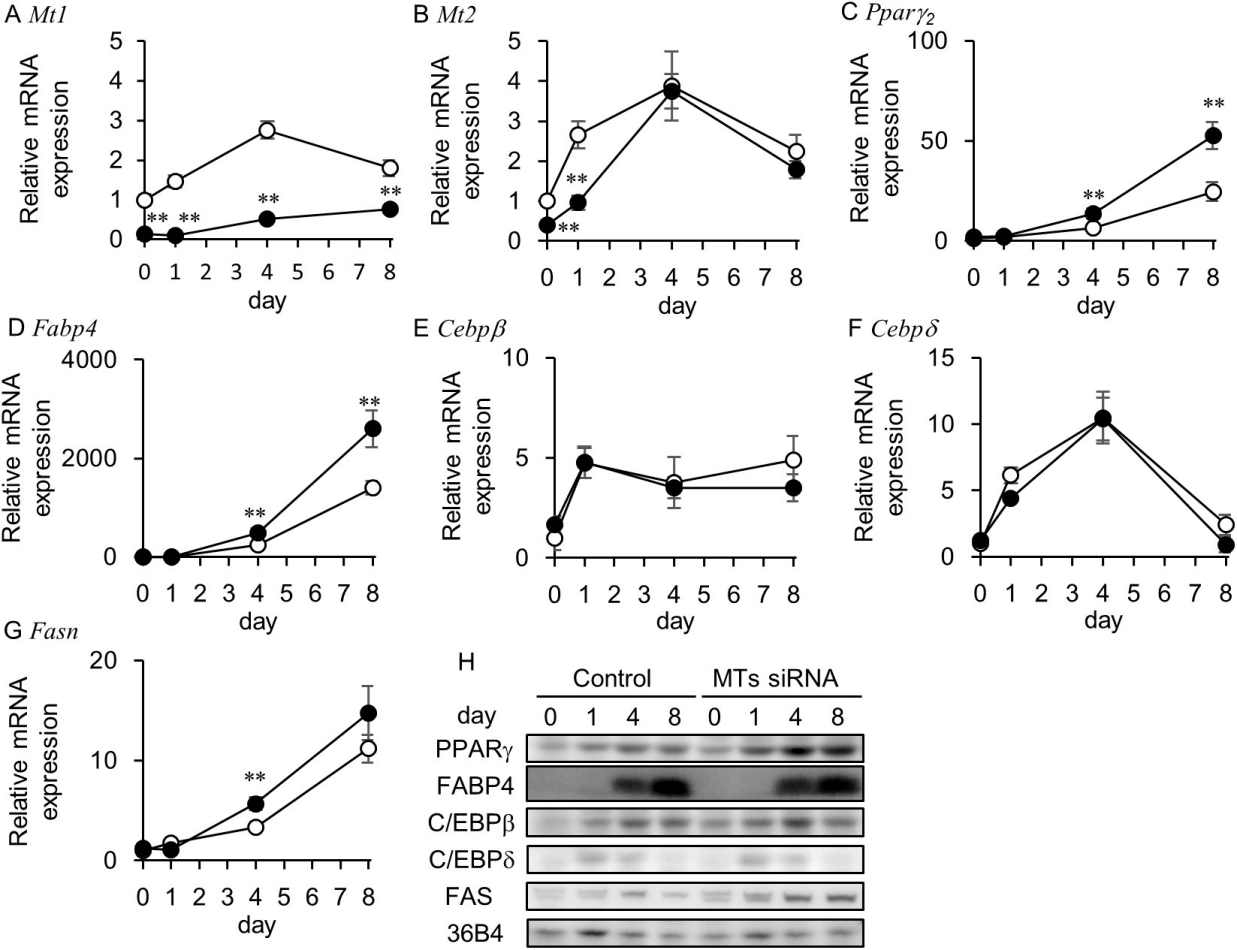
Effects of MTs siRNA on the expression of MTs and adipocyte-related genes and proteins. 3T3-L1 preadipocytes were treated with 50 nM control RNA (open circle) or MTs siRNA (closed circle) 24 h before the addition of DIM (day -1). Total RNA was isolated on day 0, 1, 4, and 8, and the expressions of (A) *mt1*, (B) *mt2*, (C) *pparγ*, (D) *fabp4*, (E) *cebpβ*, and (F) *cebpδ*, were determined using real-time PCR. All mRNA levels were normalized to the expression level of the *36B4* gene and are shown as fold induction from the control RNA-treated cells on day 0. Data are expressed as the mean ± SD (*n* = 3). ^**^P < 0.01, compared with control RNA-treated cells at the same time point. (G) Cell lysates from the control RNA-treated cells (control) and the MTs siRNA-treated cells (MTs siRNA) were obtained at the indicated points. The protein expression of PPARγ, FABP4, C/EBPβ, C/EBPδ, and FAS (with 36B4 as a loading control) during the adipogenic differentiation of 3T3-L1 cells was determined using immunoblotting.

**Fig 3:**
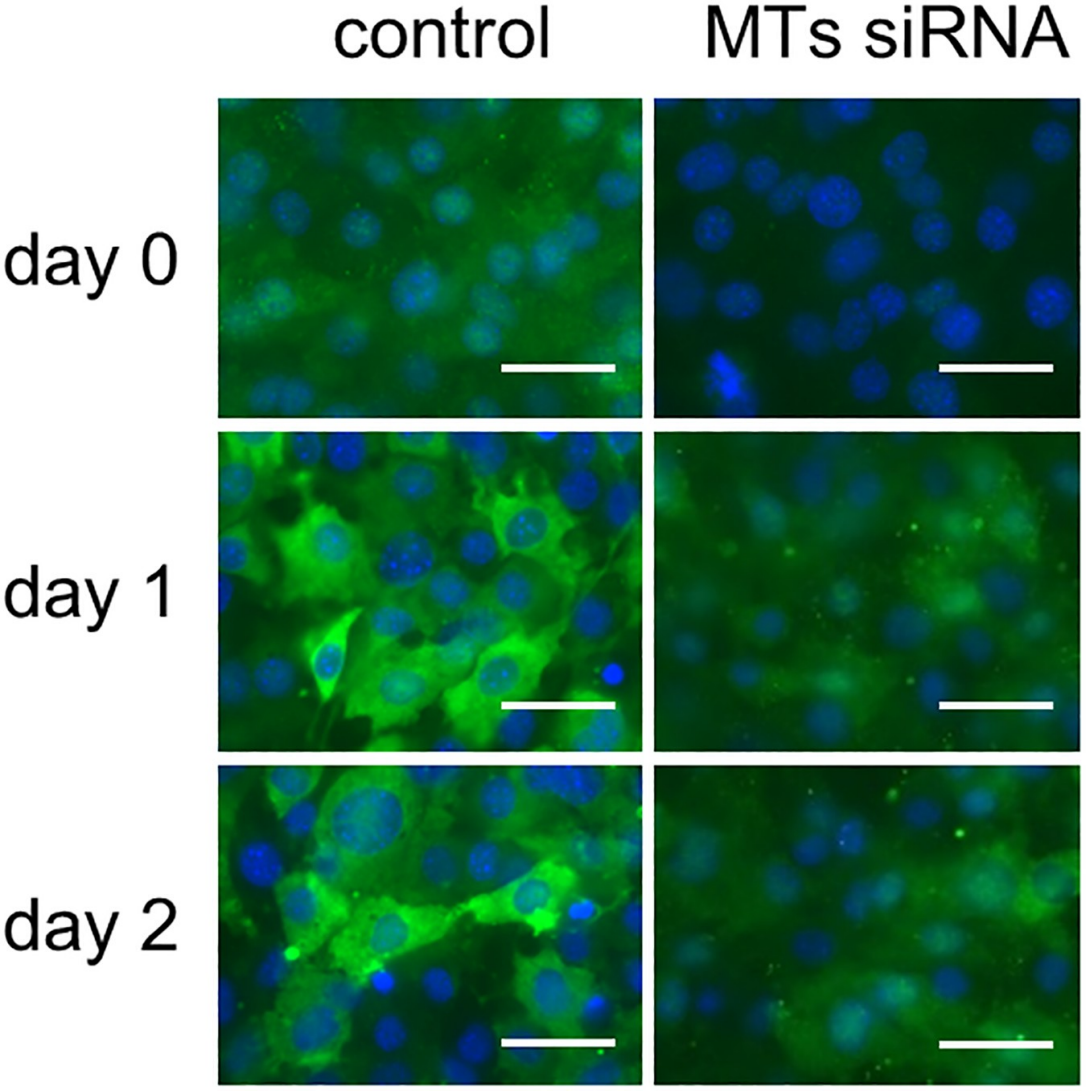
Effects of MTs siRNA on the expression of MT1 and MT2 total proteins. 3T3-L1 preadipocytes were treated with 50 nM control RNA or MTs siRNA 24 h before the addition of DIM (day -1). The cells were fixed with 4% paraformaldehyde on day 0, 1, and 2, and the expression of MT proteins was detected using a primary antibody against mouse MT1 and MT2 total proteins followed by Alexa Fluor 488-conjugated secondary antibody (Green). Nuclei were stained with Hoechst 33258 (blue). Bar = 40 μm.

**Fig 4:**
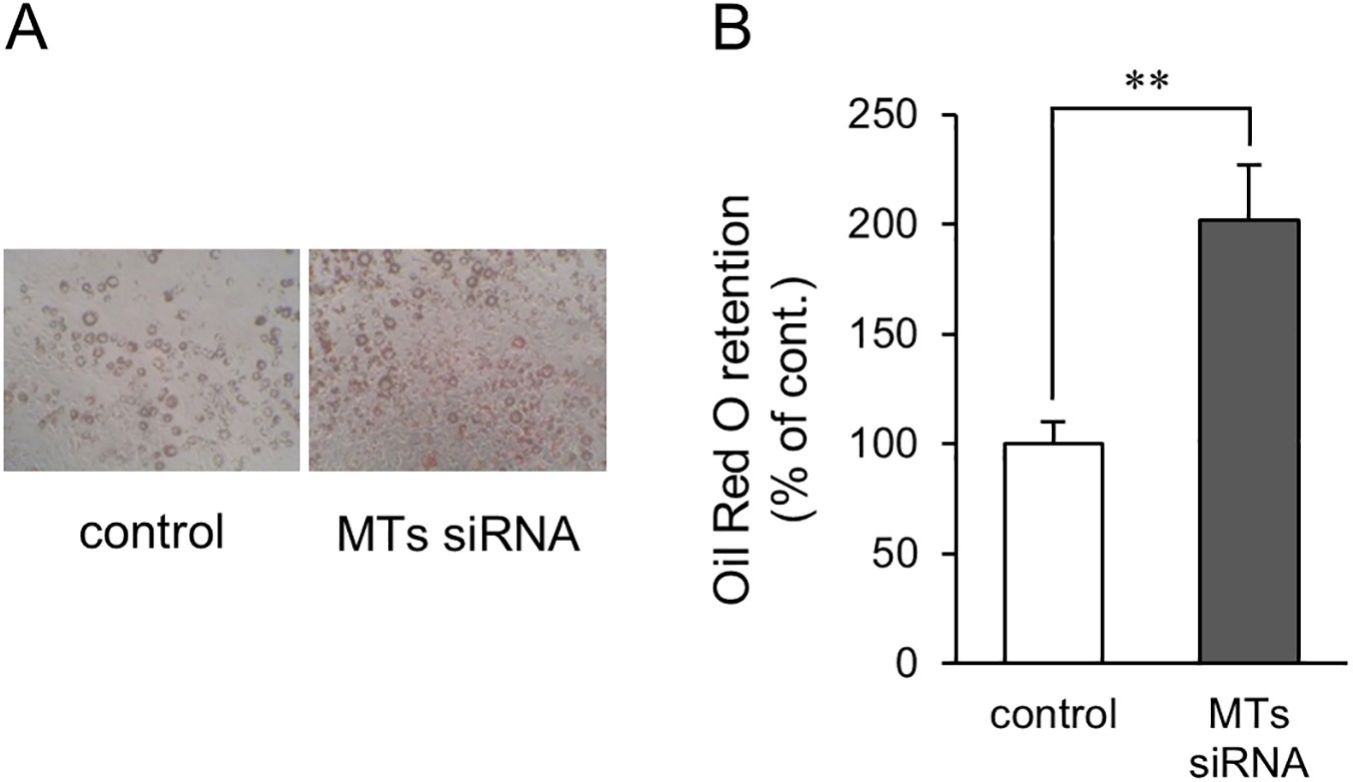
Effect of MTs siRNA on lipid accumulation in 3T3-L1 adipocytes. 3T3-L1 preadipocytes were treated with 50 nM control RNA or MTs siRNA 24 h before the induction of differentiation. On day 2, the culture medium was changed to medium supplemented with 1 μg/ml insulin. On or after day 4, the medium was replaced every 2 days. Natural lipids in the fixed cells were stained with Oil Red O. (A) Representative images of Oil Red O staining of 3T3-L1 adipocytes pretreated with control RNA (control) or MTs siRNA. (B) Absorbance of Oil Red O was measured, and Oil Red O retention is expressed as a percentage relative to the control RNA-treated cells. Data are expressed as the mean ± SD (*n* = 5). ** represents a *P* value of < 0.01.

In order to determine whether knockdown of the MT1 and MT2 genes is involved in adipocyte differentiation *in vitro*, we evaluated the ability of small interfering RNA (siRNA) to silence MT genes in the preadipocyte 3T3-L1 cell line, which is a widely used model for the study of adipocyte differentiation. The expression levels of the metallothionein genes *Mt1* (Fig 2A) and *Mt2* (Fig 2B) transiently increased, which culminated on day 4, after the induction of adipocyte differentiation. The MTs siRNA pretreatment significantly decreased the expression level of *Mt1* (Fig 2A) from day 0 to day 8 and of *Mt2* (Fig 2B) from day 0 to day 1. The intensity of indirect immunofluorescence using anti-MT1 and MT2 protein antibodies was also increased by stimulating the induction of adipogenic differentiation (Fig 3). This increased fluorescence was suppressed by the MTs siRNA pretreatment (Fig 3). On day 8 after the treatment of the DIM, we compared lipid accumulation in the control cells and the MTs siRNA-treated cells (Fig 4). The retained amount of Oil Red O (a red dye used for staining neutral lipids) was significantly higher in the MTs siRNA-treated cells than in the control RNA-treated cells (Fig 4A and 4B).

### Dexamethasone in the adipogenic differentiation medium mainly stimulates the expression of MT1 and MT2 in 3T3-L1 cells

**Fig 5:**
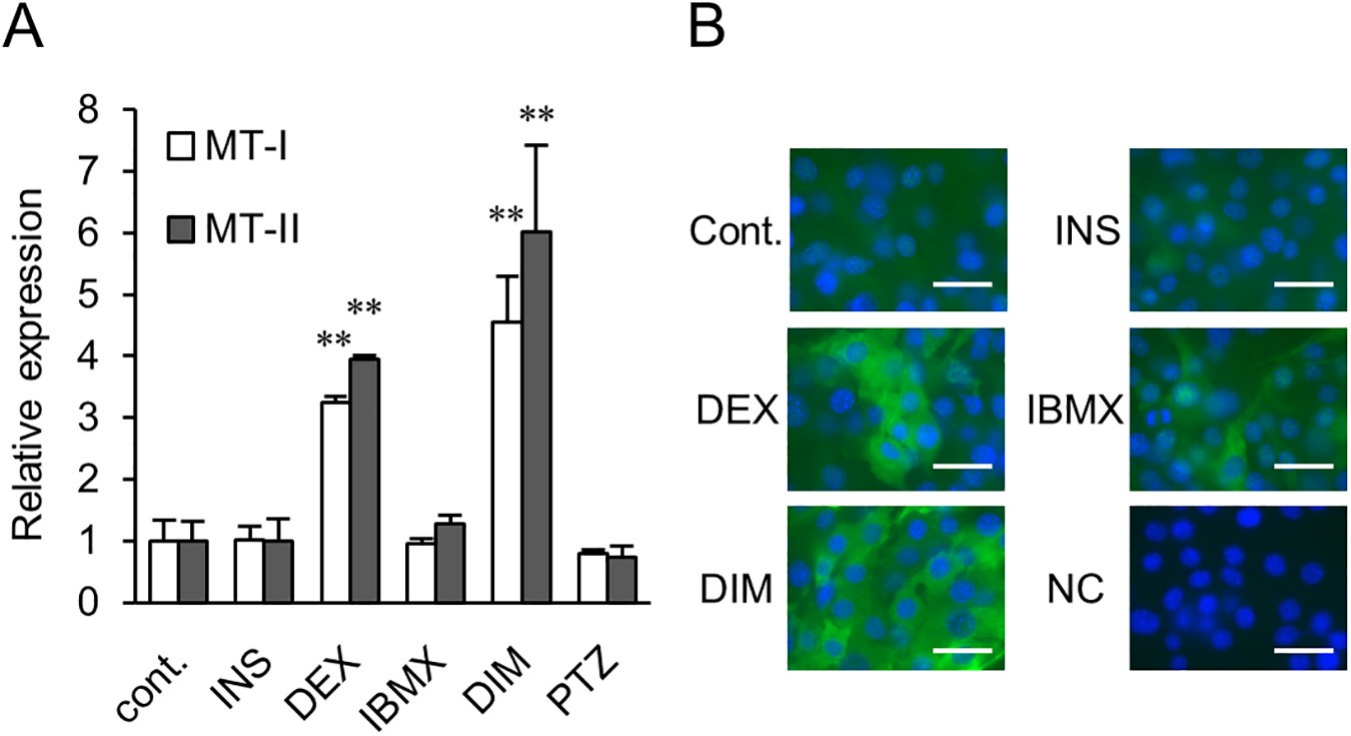
Effect of standard DIM and three separate components on MT expression. 3T3-L1 preadipocytes were treated with DIM or three components separately (1 μg/ml insulin (INS), 1 μM DEX, and 0.5 mM IBMX). A thiazolidinedione, i.e., pioglitazone (PTZ), was also used at a concentration of 1 μM. (A) The expression levels of *mt1* mRNA (open columns) and *mt2* mRNA (filled columns) are shown as percentages relative to the cells cultured in DMEM + 10% FBS (cont.). Data are expressed as the mean ± SD (*n* = 3). ^**^P < 0.01, compared with the control cells. (B) The expressions of the MT proteins were detected using anti-MT1 and MT2 total proteins followed by Alexa Fluor 488-conjugated secondary antibody (Green). Negative control (NC); staining without the primary antibody. Nuclei were stained with Hoechst 33258 (blue). Bar = 40 μm.

In order to determine which components in standard DIM for 3T3-L1 preadipocytes were required for the upregulation of MT1 and MT2 expression, 3T3-L1 preadipocytes were incubated in the presence and absence of 1 μg/ml insulin, 1 μM DEX, 0.5 mM IBMX, 1 μM pioglitazone (PTZ), a PPARγ agonist and DIM. As shown in Fig 5A, the *Mt1* and *Mt2* mRNA levels were significantly elevated by DEX stimulation. MT proteins were also upregulated by DEX treatment (Fig 5B). IBMX marginally increased *Mt2* expression but not *Mt1* expression (Fig 5A). The fluorescence intensity was also increased in the IBMX-treated cells (Fig 5B), suggesting that MT2 proteins are produced by IBMX stimulation. The insulin and PTZ treatments did not induce MT1 or MT2 expression (Fig 5A and 5B).

### Expression levels of mRNAs and proteins involved in adipocyte differentiation in MTs siRNA-pretreated 3T3-L1 cells

Quantitative RT-PCR analysis showed that the mRNA expression levels of *Pparγ*_*2*_ (Fig 2C) and *Cebpα* (S1A Fig), the master regulators of adipogenesis, *Fabp4* (Fig 2D), a genetic marker of mature adipocytes, and *Lep* (S1B Fig), the gene for leptin (an adipocyte-specific hormone) were significantly higher in the MTs siRNA-pretreated adipocytes than in the siRNA control-treated cells on day 4 and day 8. On the other hand, the MTs siRNA pretreatment did not affect the mRNA expression levels of *Cebpβ* (Fig 2E) and *Cebpδ* (Fig 2F), which are expressed in the early stage of adipocyte differentiation of 3T3-L1 and are involved in the induction of *Pparγ*_*2*_ expression. The protein expression levels of C/EBPβ, C/EBPδ, PPARγ, and FABP4 were examined by immunoblotting (Fig 2H). These protein levels correlated well with transcript levels. The expression level of *Fasn* mRNA, the gene for a major enzyme of fatty acid synthesis (fatty acid synthase, FAS), was greater in the MTs siRNA-treated group than the control group on day 4 (Fig 2G), but not on day 8. In contrast, expression levels of FAS in the MTs siRNA-treated group were higher than in the control group on day 4 and day 8 (Fig 2H).

### MT mRNA knockdown enhances DNA synthesis in mitotic clonal expansion of 3T3-L1 preadipocytes

**Fig 6:**
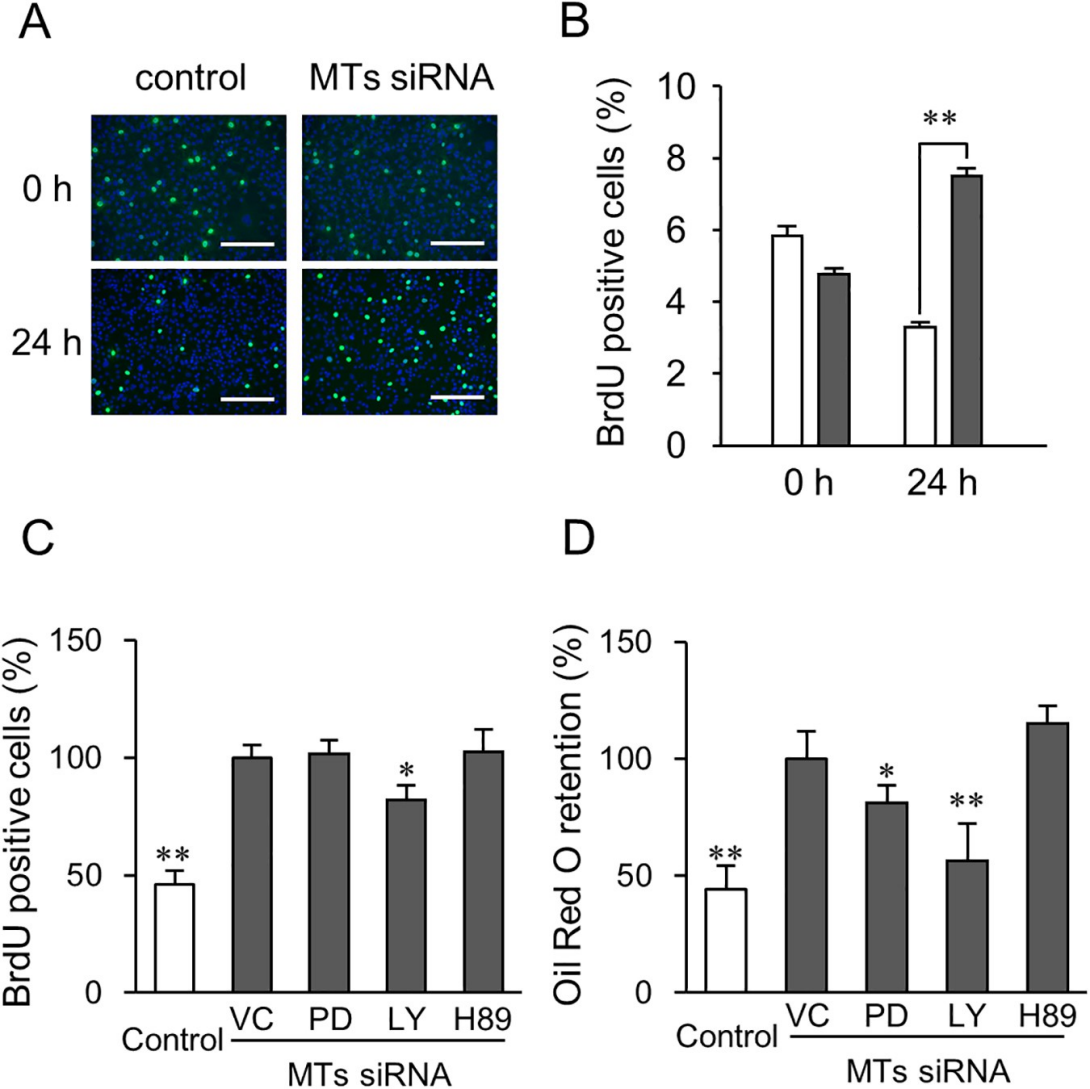
Effect of MTs siRNA on BrdU incorporation during post-confluent mitosis, known as mitosis clonal expansion. After siRNA pretreatment for 24 h, 3T3-L1 preadipocytes were stimulated with DIM for 0 or 24 h followed by exposure to 10 μM BrdU for 2 h. (A) The BrdU incorporated into replicating DNA was detected using an anti-BrdU primary antibody and fluorescein-conjugated secondary antibody using a fluorescence microscope (green). All nuclei were counterstained with Hoechest 33258 (blue). Bar = 200 μm. (B) The number of BrdU-positive cells is expressed as a percentage of Hoechst-stained nuclei. (C) and (D) After siRNA pretreatment for 24 h, 3T3-L1 preadipocytes were not treated (VC, vehicle control (DMSO)), or were treated with the indicated inhibitors (50 μM PD98059 (PD), 50 μM LY294002 (LY), or H89) for 30 min. (C) After treatment, the cells were stimulated with DIM for 24 h. The number of BrdU-positive cells is expressed as a percentage of the number of MTs siRNA-treated cells without inhibitor treatment. (D) After treatment, 3T3-L1 preadipocytes were induced to differentiate for eight days, as described in the Materials and Methods section. Natural lipids in the fixed cells were stained with Oil Red O. The absorbance of Oil Red O was measured, and Oil Red O retention is expressed as a percentage of the number of MTs siRNA-treated cells without inhibitor treatment. (B), (C), and (D) Open columns are control RNA-pretreated cells, and closed columns are MTs siRNA-pretreated cells. Data are expressed as the mean ± SD (B and C, *n* = 3; D, *n* = 5). (B) ** *P* < 0.01. (C) and (D) * *P* < 0.05 and ** *P* < 0.01, compared to MTs siRNA-treated cells without inhibitor treatment.

After DIM treatment, growth-arrested confluent 3T3-L1 preadipocytes re-enter the cell cycle, which is generally called “mitotic clonal expansion”, and undergo a few more rounds of cell division and then start the adipocyte differentiation process.[23–26] Before cell division, DNA synthesis transiently occurs in 3T3-L1 preadipocytes, which undergo mitotic clonal expansion after the addition of DIM. We focused on the effect of the MTs siRNA pretreatment on DNA synthesis during mitotic clonal expansion. DNA synthesis in proliferating cells was evaluated by the incorporation of a thymidine analog, i.e., BrdU. There was a similar number of BrdU-positive cells in the MTs siRNA- and control siRNA-pretreated cells before the DIM treatment (Fig 6A and 6B). Twenty-four hours after changing the medium to the DIM, the MTs siRNA-pretreated cells had more BrdU positive cells than the control siRNA-pretreated cells (Fig 6A and 6B).

### Mitotic clonal expansion and lipid accumulation enhanced by MT mRNA knockdown are suppressed by a phosphoinositide 3-kinase inhibitor

We next investigated the mechanism of the cell proliferation and lipid accumulation as enhanced by the MTs siRNA pretreatment. We tested the effects of inhibitors on enzymes involved in the adipocyte differentiation induced by standard DIM. Specifically, PI3K and MAPKK activated by the insulin signaling pathway were inhibited by LY294002 and PD98059, respectively, and H89 is an inhibitor against PKA activated by cyclic AMP (cAMP), which is increased by the phosphodiesterase (PDE) inhibitor IBMX. As shown in Fig 6C, the enhanced proliferation resulting from the MTs siRNA pretreatment was suppressed by LY294002, whereas neither PD98059 nor H89 were effective. The MTs siRNA-promoted lipid accumulation in the 3T3-L1 adipocytes on day 8 was significantly blocked by LY294002 (Fig 6D). PD98059 slightly decreased lipid accumulation compared with the DMSO-treated control (Fig 6D). These results suggest that adipocyte differentiation promoted by MT gene knockdown can only occur in the presence of the insulin signaling pathway.

### Insulin-stimulated phosphorylation of Akt is regulated by MT gene silencing and knockout

**Fig 7:**
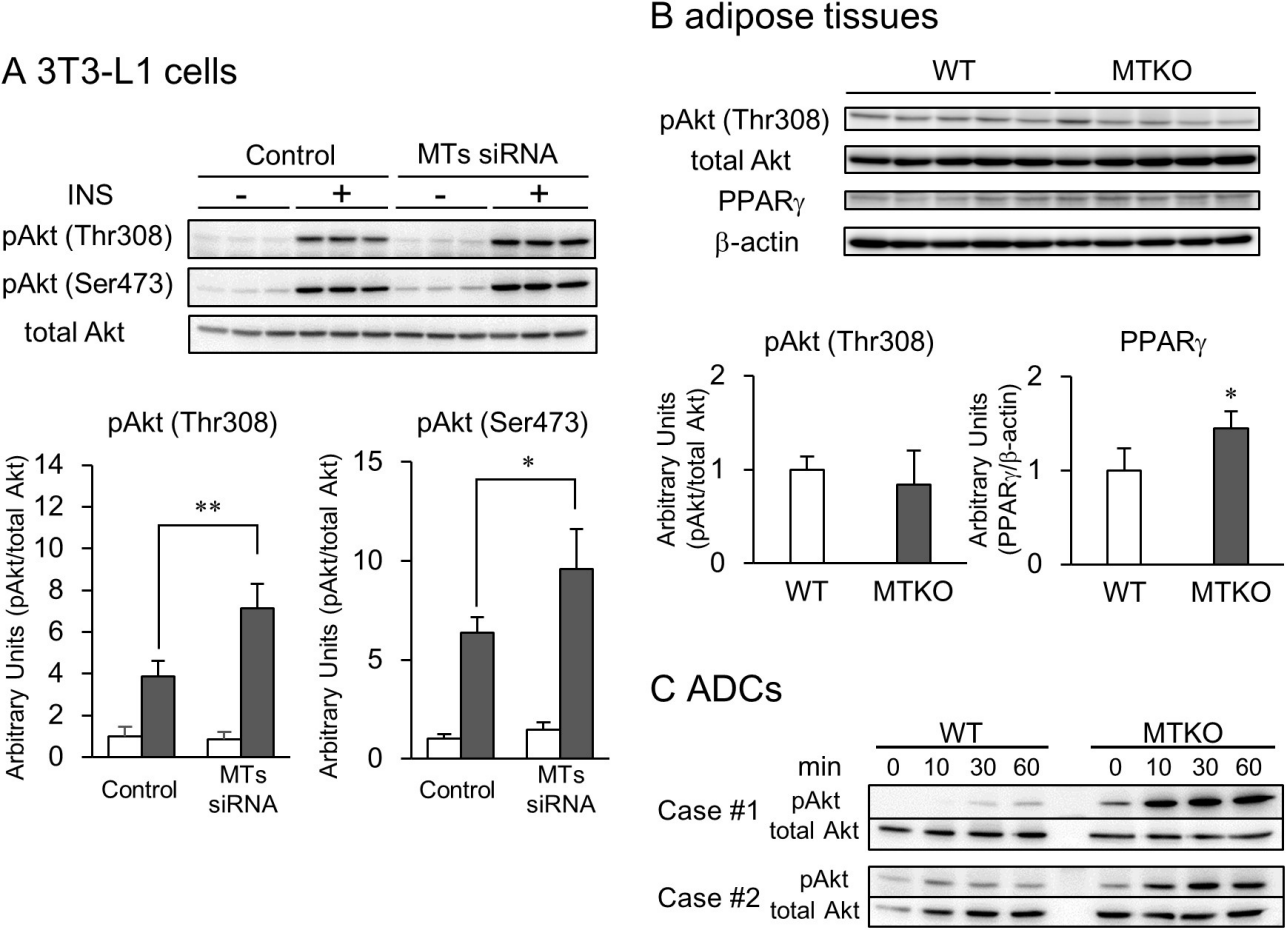
Effect of MT gene silencing and MT knockout on Akt phosphorylation. Cell lysates and tissue extracts were separated on 10% gels using SDS-PAGE. Immunoblotting was performed with the indicated antibodies. The phosphorylation ratios of Akt (pAkt) proteins or the expression ratios of PPARγ to β-actin were calculated by densitometric analyses of immunoreactive bands. (A) After siRNA pretreatment, the cells were pre-incubated with 1 μM DEX and 0.5 mM IBMX for 2 h and incubated in serum-free medium for 1 h. Then, the serum-free medium in the presence or absence of 10 nM insulin was added to the cells for 30 min. Open columns are cells untreated with insulin and closed columns are cells treated with 10 nM insulin. Data are expressed as the mean ± SD (*n* = 3). * and ** represent *P* values of < 0.05 and < 0.01, respectively. (B) Extracts from the adipose tissue of five-day-old mice were subjected to electrophoresis on SDS-PAGE gel. Open columns are WT mice and closed columns are MTKO mice. Data are expressed as the mean ± SD (*n* = 5). * represents *P* values of < 0.05. (C) ADCs derived from the adipose tissues of five-day-old WT and MTKO mice (two individuals of each genotype, Case #1 and Case #2) were incubated with 1 nM insulin in serum-free medium for the indicated time.

Insulin activates downstream Akt kinase activity, which is achieved by the phosphorylation of the Thr308 residue and Ser473 residue.[27] In order to evaluate whether MT gene silencing enhanced the insulin signaling pathway, we analyzed the activation of Akt by phosphorylation on Thr308 and Ser473 (Fig 7A) following insulin treatment. Whereas the exposure of 3T3-L1 cells to 10 nM insulin increased in phosphorylated Akt, the phosphorylation of Akt in the MTs siRNA-treated cells was more sensitive to insulin stimulation compared with the siRNA control-treated cells (Fig 7A). Since the MTs siRNA enhanced the insulin-stimulated activation of Akt in the 3T3-L1 cells, we next evaluated the phosphorylated Akt in the adipose tissue of five-day-old WT and MTKO mice. In contrast to the results of MT gene silencing *in vitro*, the adipose tissues of MTKO mice contained similar amounts of phosphorylated Akt on Thr308 as that of WT mice (Fig 7B). However, expression of the adipogenic marker PPARγ in the MTKO adipose tissue was greater than that of WT mice (Fig 7B). When MTKO and WT adipose tissue-derived preadipocytes, ADCs, were stimulated with only 1 nM insulin *in vitro*, Akt phosphorylation levels were higher in the MTKO cells (Fig 7C).

### Involvement of ROS in lipid accumulation enhanced by MT mRNA knockdown

**Fig 8:**
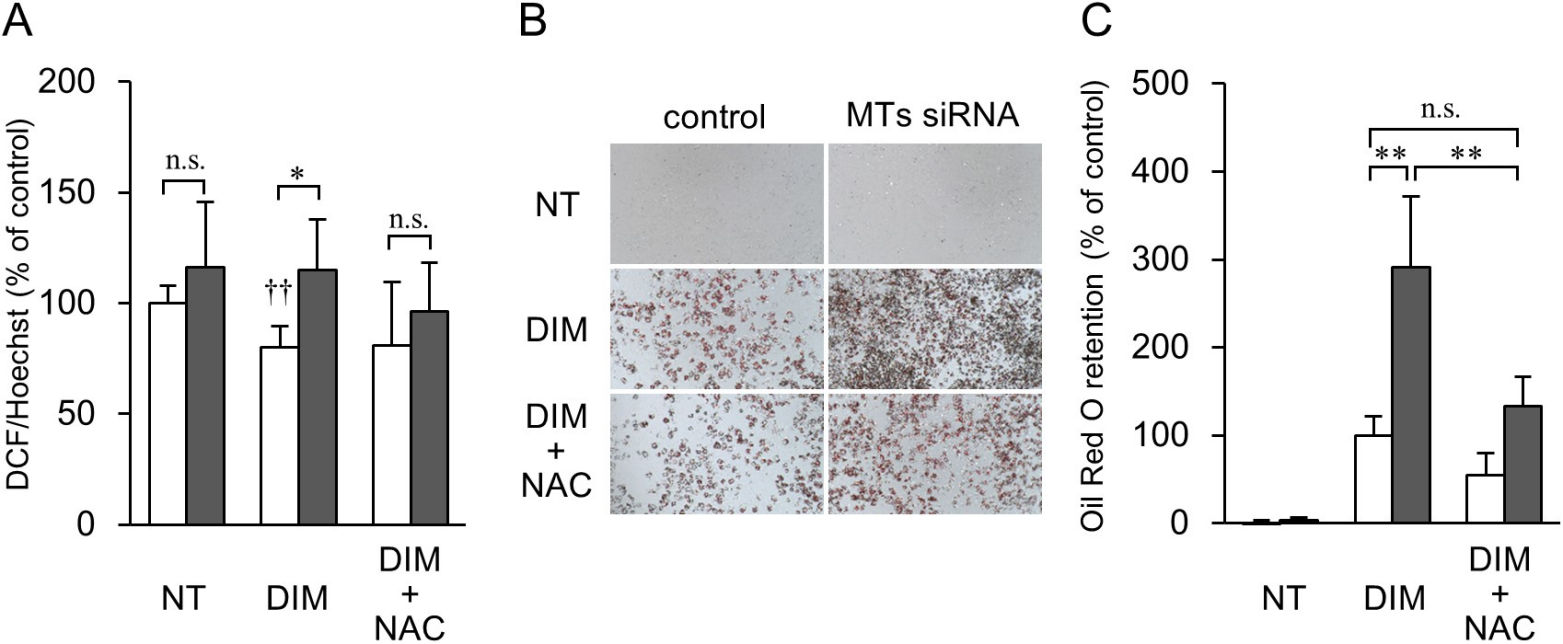
Effect of ROS on lipid accumulation in 3T3-L1 cells pretreated with MTs siRNA. (A) Production of ROS was determined using the DHFH-DA method. After pre-treatment with control RNA (open columns) or MTs siRNA (closed columns), 3T3-L1 cells were incubated in the presence or absence of 1 μM DEX, 0.5 mM IBMX, and 1 mM NAC for 1 h. Serum-free medium, including 10 μM DCFH-DA was then added to the cells for 30 min, in the presence or absence of 1 μg/mL insulin. After the cells had been fixed, ROS levels were measured as described in the Materials and Methods section. NT, non-treatment with DIM (DEX, IBMX, and insulin); DIM, treatment with DIM; DIM + NAC, treatment with DIM and 1 mM NAC. Data are expressed as the mean ± SD (*n* = 5). * and ** represent *P* values of < 0.05 and < 0.01, respectively. †† represents a *P* value of < 0.01 vs 3T3-L1 cells pretreated with siRNA control and not with DIM. n.s., not significant. (B) Representative images of Oil Red O staining in 3T3-L1 preadipocytes and adipocytes. 3T3-L1 cells were pretreated with control RNA or MTs siRNA for 24 h. Adipogenic differentiation of 3T3-L1 was then initiated with DIM (shown as DIM) or DIM and 1 mM NAC (DIM + NAC). On day 2, the culture medium was changed to medium supplemented with 1 μg/ml insulin. On or after day 4, the medium was replaced every two days. Natural lipids in the fixed cells were stained with Oil Red O. Non-differentiated 3T3-L1 preadipocytes are shown as NT. (C) Absorbance of Oil Red O was measured, and Oil Red O retention is expressed as a percentage of retention by the control RNA-treated cells. Data are expressed as the mean ± SD (*n* = 5). ** represents a *P* value of < 0.01.

ROS play an important role in the activation of the PI3K/Akt signaling pathway.[28–30] MTs have an antioxidant function, and we therefore hypothesized that MT deficiency induces a redox imbalance and then promotes adipocyte differentiation. As shown in Fig 8A, pretreatment with MTs siRNA did not change the basal ROS level in 3T3-L1 preadipocytes. The treatment with DIM decreased the ROS level in control RNA-pretreated cells, but not in MTs siRNA-pretreated cells. This difference was eliminated by an antioxidant, *N*-acetylcysteine (NAC), which was used as a substitute for antioxidant protein MTs. Pretreatment with NAC before initiating the induction of adipocyte differentiation decreased lipid accumulation in 3T3-L1 adipocytes pretreated with MTs siRNA to the level of control RNA-pretreated 3T3-L1 cells (Fig 8B and 8C).

## Discussion

In the present study, we showed that five-day-old MTKO mice already had greater fat mass compared with wild type mice (Fig 1), suggesting that MTs regulate adipose tissue formation in mice during the lactation stage. There was no difference in WAT weight between adult WT and MTKO mice.[16] Although this earlier adipose development in MTKO mice had little influence on body weight gain and could not induce obesity under standard diet conditions, HFD feeding may result in obesity through well-developed fat associated with MT deficiency in infancy. In this study, we showed that MTs siRNA treatment increased the expression levels of adipocyte-specific genes and proteins, such as PPARγ and FABP4, as well as a protein involved in fatty acid synthesis (Fig 2), and enhanced lipid accumulation in 3T3-L1 adipocytes (Fig 4), suggesting that MTs negatively control the differentiation of preadipocytes into adipocytes. These *in vitro* results are consistent with the earlier development of adipose tissues of MTKO mice *in vivo*.

Transient elevation of the MT expression level has been observed in 3T3-L1 cells after stimulation with DIM.[31,32] MTs were induced by treatment with dexamethasone and activators of the cAMP/PKA pathway, i.e., forskolin and bromo-cAMP, in rat adipocytes.[15,33] MT expression in rat liver was also regulated by cAMP and glucocorticoids.[34] Similar to these results, we showed that dexamethasone mainly upregulated MT expression (Fig 5). The transcription of MT genes is known to be induced by glucocorticoids through glucocorticoid response elements (GREs) in the promoter region.[4,35] A PDE inhibitor, i.e., IBMX, which can result in activation of cAMP/PKA signaling, faintly regulated MT expression in the differentiation of 3T3-L1 cells. Insulin and pioglitazone did not influence the transcription of MT genes in 3T3-L1 cells (Fig 5).

Insulin is a peptide hormone that plays a role in the regulation of blood glucose levels and is also a potent adipogenic regulator governing differentiation of preadipocytes into mature adipocytes.[23] Akt is a well-known key mediator of cell cycle progression and cell survival in insulin signaling.[36,37] The downregulation of Akt activity contributes to inhibition of clonal expansion in 3T3-L1 preadipocytes.[38] In the present study, we demonstrated that the MTs siRNA pretreatment activated Akt proteins induced by insulin treatment in 3T3-L1 preadipocytes (Fig 7), and 3T3-L1 adipocytes with lipid droplets increased by MTs siRNA were suppressed by the PI3K inhibitor LY294002 (Fig 6). Haynes *et al*. showed that MT treatment inhibited insulin-stimulated glucose uptake by 3T3-L1 adipocytes.[39] Fu *et al*. reported that MT1G suppressed thyroid cancer cells by inhibiting the PI3K/Akt signaling pathway.[40] Recently, Summermatter *et al*. showed that suppressed expression of MT1 and MT2 genes was associated with activation of the Akt pathway, which is a crucial regulator of skeletal muscle hypertrophy, and an increase in myotube fiber size, both *in vitro* and *in vivo*. [41] Therefore, MTs may negatively regulate the adipogenic differentiation of 3T3-L1 cells via inhibition of the insulin-Akt pathway. Whereas the adipose tissue of MTKO mice was well-developed (Fig 1) and had highly expressed PPARγ (Fig 7B), we did not observed higher Akt phosphorylation than in WT mice at five days old (Fig 7B). On the other hand, ADCs obtained from MTKO mice were more sensitive to insulin stimulation than those of WT mice *in vitro* (Fig 7C). These results suggested that preadipocytes in the adipose tissue of MTKO mice are more sensitive, in terms of Akt phosphorylation, to insulin stimulation than those of WT mice. However, the adipose tissues of MTKO mice may be more developed than those of WT mice at five days old, and Akt activation in these tissues may thus occur at an earlier stage, as a local response in pre-adipose tissue.

**Fig 9:**
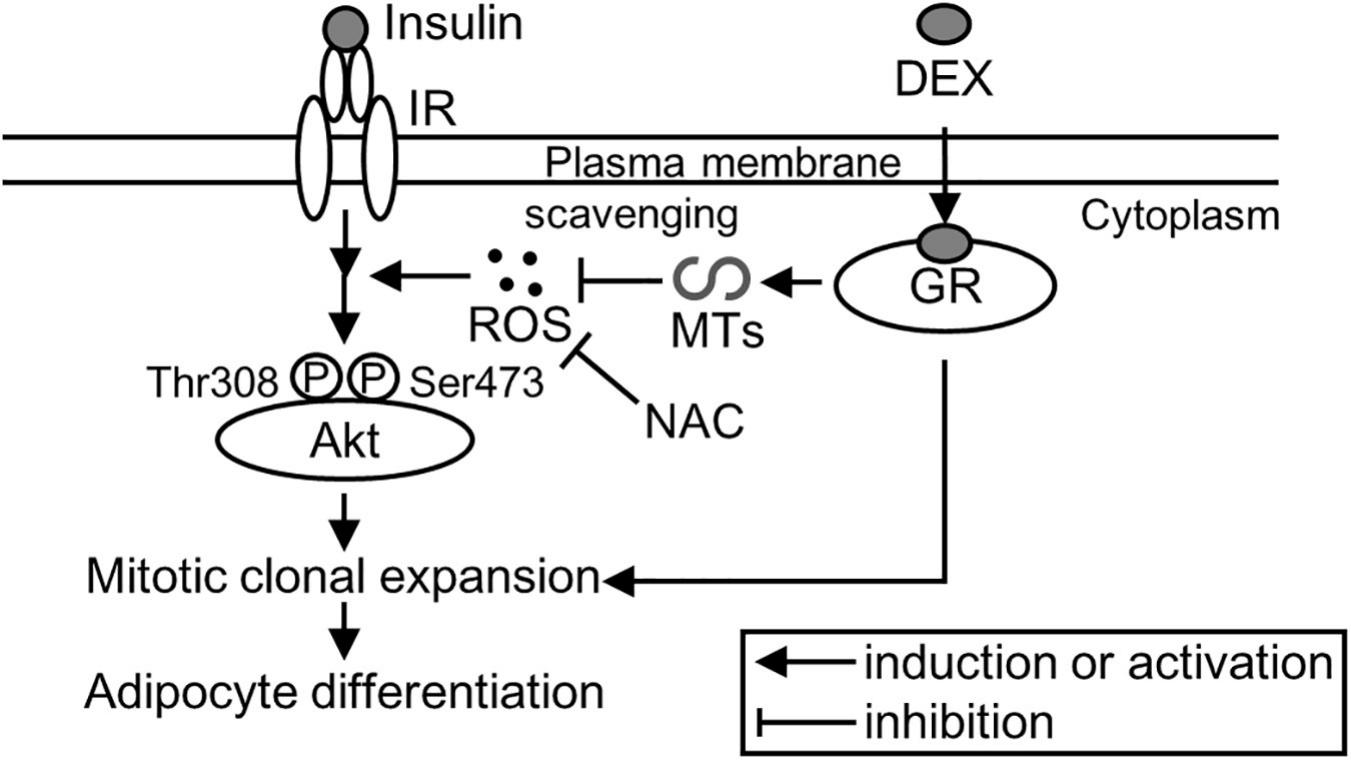
Scheme of a potential mechanism for the regulation of the insulin/Akt signaling pathway by MTs. The adipogenic differentiation of 3T3-L1 preadipocytes into adipocytes is induced with differentiation-inducing medium (DIM) containing insulin, dexamethasone (DEX), and IBMX. ROS activate insulin/Akt signaling and promote mitotic clonal expansion. Metallothioneins (MTs), which are induced by DEX treatment, may regulate insulin-Akt signaling by scavenging the ROS. IR, insulin receptor; GR, glucocorticoid receptor.

MTs siRNA enhanced DNA synthesis in mitotic clonal expansion of 3T3-L1 preadipocytes (Fig 6A and 6B). This effect was suppressed by LY294002 (Fig 6C). ROS can facilitate adipocyte differentiation by accelerating mitotic clonal expansion.[42] In addition, ROS can act as signaling molecules and modifiers of the activity of several enzymes involved in the PI3K/Akt signaling pathway. [28–30] Although MTs have an antioxidant function, basal expression levels of MTs did not affect ROS levels in 3T3-L1 cells (Fig 8A). Treatment with DIM reduced ROS levels in control RNA-pretreated cells, but not in MTs siRNA-pretreated cells (Fig 8A), suggesting that MTs, which were induced by DEX, may scavenge the ROS. NAC, which was used as a substitute for MTs, suppressed the lipid accumulation that was enhanced by MTs gene silencing. Based on this, we present a proposed scheme for the involvement of MTs in the differentiation of preadipocytes into adipocytes (Fig 9). However, MTs conversely protect against cardiomyopathy, renal injury, and burn sepsis, through Akt activation as survival signaling.[43–45] The complicated functions of MTs may depend on their expression levels, cell type, and the intracellular environment, including metal ion concentration and redox valence. In addition, because it seems that MTs siRNA affected *Mt2* expression for only 1–2 days after transfection in this study (Fig 2B), the contribution of MTs siRNA could be evaluated for only the initial stage of the differentiation of 3T3-L1 cells, and not for later stages. For instance, although there was no difference in the expression levels of *Fasn* mRNA between the siRNA-treated and the control groups on day 8 (Fig 2G), the FAS expression level was higher in the siRNA-treated group than in the control group on the same day. In this study, the efficacy for siRNA transfection via a lipofection-based method was affected by the rate of cell division. Because 3T3-L1 adipocytes are non-dividing cells, the transfection was not sufficiently effective to detect gene silencing. Further studies are thus needed to elucidate the potential role of MTs in adipocyte differentiation at later stages in the tissue development, as well as the activation of the Akt/PI3K pathway.

Although we demonstrated that female MTKO mice that were fed a HFD became obese and developed hyperleptinemia, they did not exhibit higher blood glucose or insulin levels compared with wild type mice.[16] Chen *et al*. recently reported that MT transgenic mice showed impaired glucose-stimulated insulin secretion, which promoted the development of diabetes.[46] This information suggests that MT expression negatively regulates glucose metabolism/insulin signaling and that MT deficiency enhances insulin sensitivity. It was reported that in humans, insulin-sensitive adiposity is associated with a relatively lower risk of diabetes compared with insulin-resistant adiposity.[47] Therefore, the obesity of the MTKO mice may not be more severe than metabolic disorders. However, MTKO mice have shorter lifespans than WT mice.[48] In many organisms, the insulin/insulin-like growth factor-1 signaling (IIS) pathway is a well-known conserved pathway that regulates aging, and reduction in IIS activity has been observed to lead to extended lifespans.[49] In addition, MT expression is increased in long-lived mutants.[50,51] MT deficiency in mice may accelerate aging though activation of the IIS pathway. Further multidimensional studies are needed to understand the roles of MTs in the pathophysiology of obesity, type 2 diabetes, and aging.

## Supporting information

S1 Table Primer information. Primer sets used for the RT-PCR assay.
(PDF)

S1 Fig Effects of MTs siRNA on the expression of *Cebpa* and *Lep* during the differentiation of 3T3-L1 preadipocytes into adipocytes. 3T3-L1 preadipocytes were treated with 50 nM control RNA (open circle) or MTs siRNA (closed circle) 24 h before the addition of DIM (day -1). Total RNA was isolated on day 0, 1, 4, and 8, and the expressions of (A) *Cebpa* and (B) *Lep* were determined using real-time PCR. All mRNA levels were normalized to the expression level of the *36B4* gene and are shown as fold induction from the control RNA-treated cells on day 0. Data are expressed as the mean ± SD (*n* = 3). ^**^P < 0.01, compared with control RNA-treated cells at the same time point.
(TIF)
