# Supplementary material for: Metallothioneins regulate the adipogenic differentiation of 3T3-L1 cells via the insulin signaling pathway
Source: PLoS One. 2017 Apr 20;12(4):e0176070. doi: 10.1371/journal.pone.0176070 (PMC5398611; doi:10.1371/journal.pone.0176070)
Supplement: S1 Table — Primer sets used for the RT-PCR assay. (PDF) [file pone.0176070.s001.pdf]

S1 Table. Primer information

Gene	Primer sequence		GenBank at NCBI Accession #
	Forward	Reverse	
Cebpα	5'-TGGACAAGAACAGCAACGAGTA-3'	5'-GCAGTTGCCATGGCCTTGA-3'	[NM_007678]
Cebpβ	5'-GGGGTTGTTGATGTTTTTGG-3'	5'-CGAAACGGAAAAGGTTCTCA-3'	[NM_009883]
Cebpδ	5'-GGAACACGGGAAAGCATGA-3'	5'-GGGTTAAGCCCGCAAACATTA-3'	[NM_007679]
Fabp4	5'-ATGAAATCACCGCAGACGAC-3'	5'-TTT CCATCCCACTTCTGCAC-3'	[NM_024406]
Fasn	5'-CCTGGATAGCATTCCGAACCT-3'	5'-AGCACATCTCGAAGGCTACACA-3'	[NM_007988]
Lep	5'-CCAAAACCCTCATCAAGACC-3'	5'-TCATTGGCTATCTGCAGCAC-3'	[NM_008493]
Mt1	5'-AATGTGCCCAGGGCTGTGT-3'	5'-GCTGGGTTGGTCCGATACTATT-3'	[NM_013602]
Mt2	5'-TGTGCCTCCGATGGATCCT-3'	5'-GCAGCCCTGGGAGCACTT-3'	[NM_008630]
Pparγ2	5'-TGGAATTAGATGACAGTGACTTGG-3'	5'-CTCTGTGACGATCTGCCTGAG-3'	[NM_011146]
36b4	5' -TGGGCATCACCACGAAAATC-3'	5'-TTCAGCATGTTCAGCAGTGTGG-3'	[NM_007475]
