# Supplementary figures and images for: Metallothioneins regulate the adipogenic differentiation of 3T3-L1 cells via the insulin signaling pathway
Source: PLoS One. 2017 Apr 20;12(4):e0176070. doi: 10.1371/journal.pone.0176070 (PMC5398611; doi:10.1371/journal.pone.0176070)

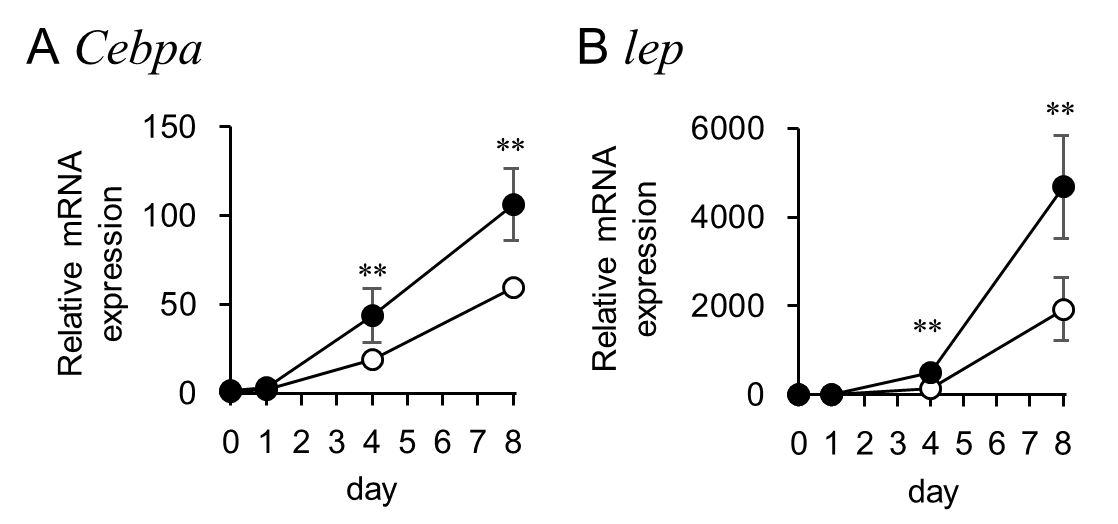

Supplement: S1 Fig — 3T3-L1 preadipocytes were treated with 50 nM control RNA (open circle) or MTs siRNA (closed circle) 24 h before the addition of DIM (day -1). Total RNA was isolated on day 0, 1, 4, and 8, and the expressions of (A) Cebpa and (B) Lep were determined using real-time PCR. All mRNA levels were normalized to the expression level of the 36B4 gene and are shown as fold induction from the control RNA-treated cells on day 0. Data are expressed as the mean ± SD (n = 3). **P < 0.01, compared with control RNA-treated cells at the same time point. (TIF) [file pone.0176070.s002.TIF]
